# Multiple disulfide‐bonded states confer extensive conformational diversity in fibrinogen

**DOI:** 10.1002/pro.70558

**Published:** 2026-04-06

**Authors:** Aster E. Pijning, Diego Butera, Philip J. Hogg

**Affiliations:** ^1^ School of Life Sciences, University of Technology Sydney Faculty of Science Sydney New South Wales Australia; ^2^ Centenary Institute University of Sydney Sydney New South Wales Australia

**Keywords:** AlphaFold, cysteine, disulfide bond, fibrin, fibrinogen

## Abstract

Disulfide bonds constrain the polypeptide backbone and reduce conformational variability in proteins. The blood clotting protein fibrinogen is constitutively produced as multiple partially disulfide‐bonded states, suggesting that individual fibrinogen molecules have a variety of conformational forms. This hypothesis was tested by resolving fibrinogen molecules on beads coated with different fibrinogen ligands and measuring their disulfide states by differential cysteine alkylation and mass spectrometry. Polyclonal anti‐fibrinogen antibodies resolved states where all 11 measured disulfides across the molecule were significantly less formed. In contrast, the GHRP peptide, which binds the fibrinogen β‐nodule, resolved states in which seven β‐nodule and central E region disulfides were significantly more formed, while the GPRP peptide, which binds the γ‐nodule, resolved states in which only one disulfide was significantly more formed. To probe the link between disulfide state and conformation, in silico analysis of all 32 possible disulfide‐bonded states of the β‐nodule revealed that the conformational flexibility of this domain and predicted GHRP interactions in its binding pocket are predicated on the oxidation state of one of the five β‐nodule disulfides, βC424–βC437. These findings indicate that the different disulfide‐bonded states of fibrinogen adopt an extensive array of conformations that are selectively recognized by different fibrinogen ligands.

## 1 INTRODUCTION

Several proteins are constitutively produced as multiple partially disulfide‐bonded states; that is where individual molecules have variable numbers of intact disulfide bonds. Both secreted soluble (Butera et al., 2014; Butera et al., 2023; Butera et al., 2025; Butera & Hogg, 2020) and intrinsic membrane (Dupuy et al., 2023; Florido et al., 2021; Pijning et al., 2021; Pijning et al., 2022) proteins are produced in this way and the indications are that this is the norm rather than the exception.

**FIGURE 1:**
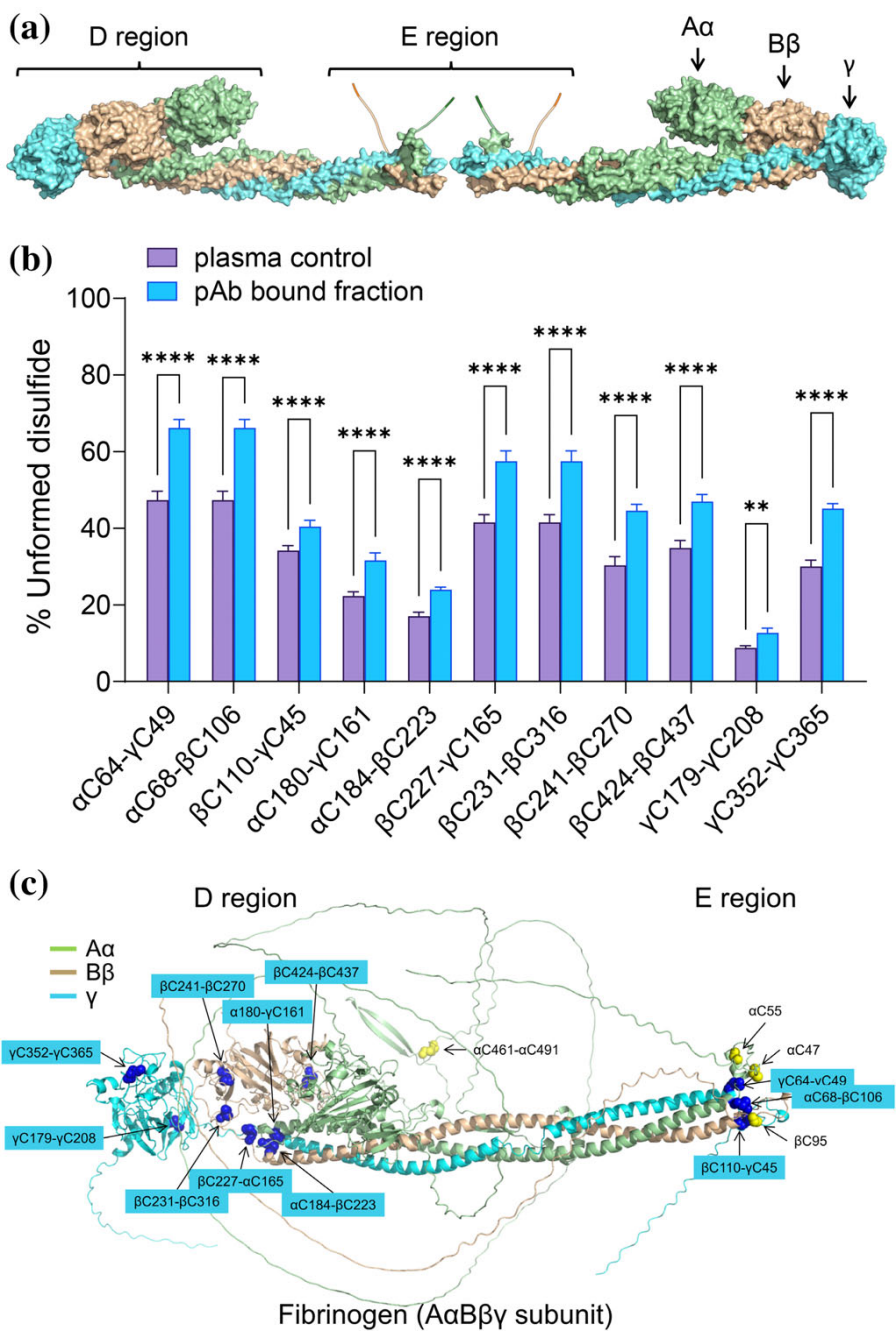
Polyclonal antibodies selectively bind fibrinogen states with globally more unformed disulfide bonds. (a) Surface representation of fibrinogen consisting of two sets of three polypeptide chains, Aα (light green), Bβ (wheat), and γ (cyan), linked head‐to‐head to form a structure with a central E region and two terminal D regions. Schematic representations of the fibrinopeptides of the Aα and Bβ chains are shown. (b) Redox states of 11 of the 12 plasma fibrinogen disulfide bonds captured on anti‐fibrinogen polyclonal antibody‐coated beads. The bars and errors represent the mean ± SD of four different healthy human plasma samples. Two‐way ANOVA with Tukey's multiple comparison tests were performed. **p < 0.01, ****p < 0.0001. (c) The AlphaFold3 ribbon structure of the fibrinogen subunit. The α chain is light green, β chain is wheat and γ chain is cyan. The 12 intrachain and interchain disulfide bonds are indicated as yellow spheres. The 11 measured bonds that are more unformed in fibrinogen states captured by polyclonal antibodies are indicated in blue.

Fibrinogen was the first example of such a protein (Butera & Hogg, 2020). It is a plasma glycoprotein and soluble precursor of fibrin that polymerizes at sites of vascular injury to stabilize the developing thrombus (Weisel & Litvinov, 2017; Wolberg, 2023). The molecule consists of two sets of three polypeptide chains, Aα, Bβ, and γ, linked head‐to‐head to form a structure with a central E region and two terminal D regions (Figure 1a). Conversion of fibrinogen to fibrin is mediated by thrombin cleavage of fibrinopeptides A and B from the N‐termini of the Aα and Bβ chains, respectively. Removal of the fibrinopeptides exposes GPR and GHRP “knobs” that bind to complementary “holes” in the γ‐ and β‐nodules of adjacent molecules to initiate fibrin fiber formation.

The fibrinogen Aα, Bβ, and γ chains contain 14 intra‐ or inter‐chain disulfide bonds located in the central E and end D regions and three additional inter‐subunit bonds that link together the two sets of chains to form the complete molecule (Table S1). Thirteen disulfide bonds (five in the E region and eight in the D region) in healthy donor plasma fibrinogen exist in formed or unformed states in different molecules of the populations (Butera & Hogg, 2020). The bonds range from 10% to 50% unformed with little donor‐to‐donor variation (Butera & Hogg, 2020).

Disulfide bonds constrain the polypeptide backbone and the absence of one or more of these covalent linkages is expected to result in a population of molecules with small to large conformational variations. Outstanding questions are the number of fibrinogen disulfide‐bonded states that are produced and evidence for their conformational differences. These questions were examined in this study by resolving fibrinogen molecules on beads coated with different fibrinogen ligands and measuring their disulfide states by differential cysteine alkylation and mass spectrometry. To test the link between disulfide state and conformation, the fibrinogen β‐nodule contains five disulfide bonds and in silico analysis of all 32 possible disulfide‐bonded states was performed.

## 2 RESULTS

The redox states of 11 disulfide bonds in the total fibrinogen pool from healthy donor human plasma were analyzed by differential cysteine alkylation using a pair of isotopic 2‐iodo‐N‐phenylacetamide alkylators and mass spectrometry (Butera et al., 2018; Butera & Hogg, 2020; Pijning et al., 2021). Plasma fibrinogen was resolved on SDS‐PAGE and the fibrinogen band collected for analysis (Figure S1). The redox states of fibrinogen were determined from the relative abundance of cysteine‐containing peptides labeled with the different isotopic alkylators. The results are expressed as percentage of the fibrinogen disulfide that is unformed in the population of molecules. The criteria we applied for analysis of disulfide bond state were matching ^12^C‐IPA‐ and ^13^C‐IPA‐labeled peptides with area under the curve (AUC) values >100,000. If the peptides reporting on the disulfide bond cysteines fell below this value they were not included in the analysis.

### 2.1 Disulfide bond formation in fibrinogen is conditional

If we consider a situation where a protein contains *n* disulfide bonds and their unformed or formed state is independent of each other, a maximum of 2^*n*^ disulfide‐bonded states are possible. For the 11 determined bond states in fibrinogen, this equates to 2048 possible disulfide‐bonded states.

For the general case for any number of disulfides, the probability that a given number of disulfide bond(s) will be formed in a protein, assuming there is no special dependence in formation of bonds, is *P*(*n*) = 2^−*n*^ (Hogg, 2020). For the 11 disulfide bonds in plasma fibrinogen, the probability that all bonds are formed, assuming no special dependencies, is 0.00049. The probability of a state containing any number of bonds is the product of the probabilities for each of the bonds (Hogg, 2020). For the 11 experimental values of mean formed state of each fibrinogen disulfide (Figure 1b, Table S2), this equates to a probability of 0.01164. The 24‐fold difference in the unconditional value of 0.00049 and the experimentally determined value of 0.01164 indicates that disulfide bond formation in fibrinogen is conditional.

The abundance of fibrinogen disulfide‐bonded states was examined by resolving plasma fibrinogen molecules on beads coated with three different fibrinogen ligands and measuring their disulfide states. Fibrinogen was in molar excess of the ligands and the reactants were mixed for 1 h so that the preferred binders were selected from the fibrinogen pool.

### 2.2 Polyclonal antibodies selectively bind fibrinogen states with globally more unformed disulfide bonds

The redox states of 11 intrachain and interchain disulfide bonds in plasma fibrinogen molecules captured on anti‐fibrinogen polyclonal antibody‐coated beads were determined. Notably, all 11 bonds across the subunit were significantly more unformed in the antibody‐bound molecules (Figure 1). For example, the αC64–γC49 and αC68–βC106 bonds in the central E region bonds are unformed in ~1 in two molecules of the plasma fibrinogen pool and unformed in ~2 in three molecules of the antibody‐bound molecules. Also, the βC241‐βC270 bond in the β‐nodule and the γC352‐γC365 bond in the γ‐nodule are unformed in ~1 in three molecules of the plasma fibrinogen pool and unformed in ~1 in two molecules of the antibody‐bound molecules.

The polyclonal antibody recognizes epitopes across the subunit. We compared these findings with the fibrinogen states that are captured by peptide ligands that bind specifically to either the β‐nodule or γ‐nodule (Figure S3). The GHRP and GPRP peptides mimic the B:b and A:a knob hole interaction in protofibril fibrin formation, respectively.

### 2.3 A β‐nodule ligand selectively binds fibrinogen states where β‐nodule and central E region disulfides are more formed

**FIGURE 2:**
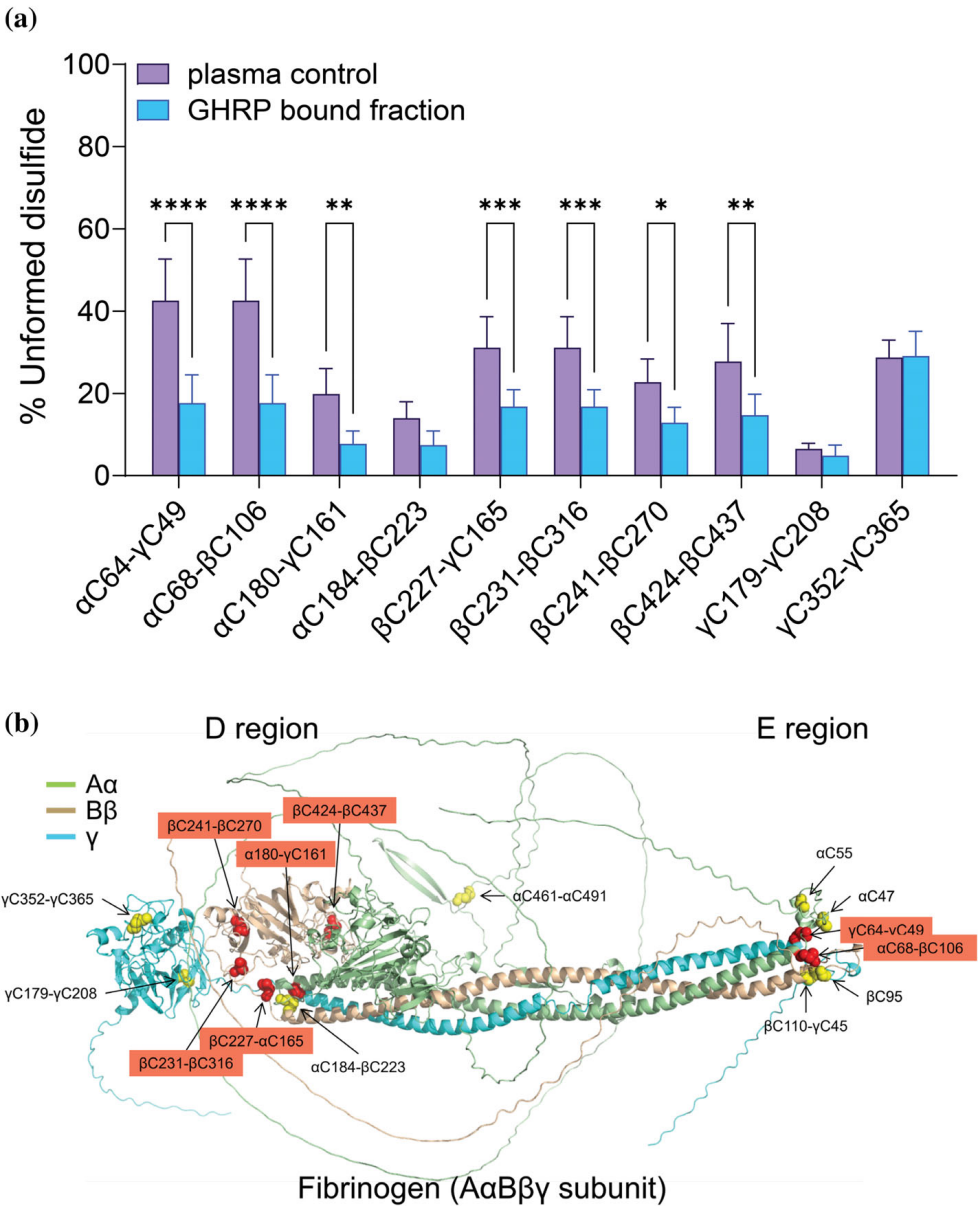
A β‐nodule ligand selectively binds fibrinogen states where β‐nodule and central E region disulfides are more formed. (a) Redox states of 10 of the 12 plasma fibrinogen disulfide bonds captured on GHRP‐coated beads. The bars and errors represent the mean ± SD of six different healthy human plasma samples. Two‐way ANOVA with Tukey's multiple comparison tests were performed. *p < 0.05, **p < 0.01, ***p < 0.001, ****p < 0.0001. (b) The AlphaFold3 ribbon structure of the fibrinogen subunit. The α chain is light green, β chain is wheat and γ chain is cyan. The 12 intrachain and interchain disulfide bonds are indicated as yellow spheres. The 10 measured bonds that are more formed in fibrinogen states captured by GHRP peptide are indicated in red.

The redox states of 10 intrachain and interchain disulfide bonds in plasma fibrinogen molecules captured on GHRP‐coated beads were determined. Seven β‐nodule and central E region disulfides were significantly more formed, which contrasts with the results using the polyclonal antibody ligand (Figure 2). For example, the αC64–γC49 and αC68‐βC106 bonds in the central E region are unformed in ~1 in two molecules of the plasma fibrinogen pool and ~1 in five molecules of the GHRP‐bound molecules. The β‐nodule βC227–γC165, βC231–βC316, and βC424–βC437 bonds, for instance, are unformed in ~1 in three molecules of the plasma fibrinogen pool and ~1 in five molecules of the GHRP‐bound molecules.

The preferential engagement of GHRP with β‐nodules with more formed disulfides could be a result of selection of β‐nodules with more formed disulfides from the total fibrinogen pool or formation of nodule disulfides triggered by peptide binding. To distinguish these possibilities, plasma was incubated with saturating concentrations of GHRP peptide and effects on fibrinogen disulfide states were measured. GHRP binds to the β‐nodule with a dissociation constant (*K*_d_) of 140 μM (Weisel & Litvinov, 2013). Incubation of pooled plasma with 2‐, 5‐, or 10‐times *K*_d_ concentrations of GHRP for 1 h had no effect on the redox states of fibrinogen disulfide bonds (Figure S4). This result indicates that the peptide does not mediate formation of the disulfides; rather, GHRP selects fibrinogen molecules with more formed disulfides in the β‐nodules and E region.

### 2.4 A γ‐nodule ligand does not select for different disulfide‐bonded fibrinogen states

**FIGURE 3:**
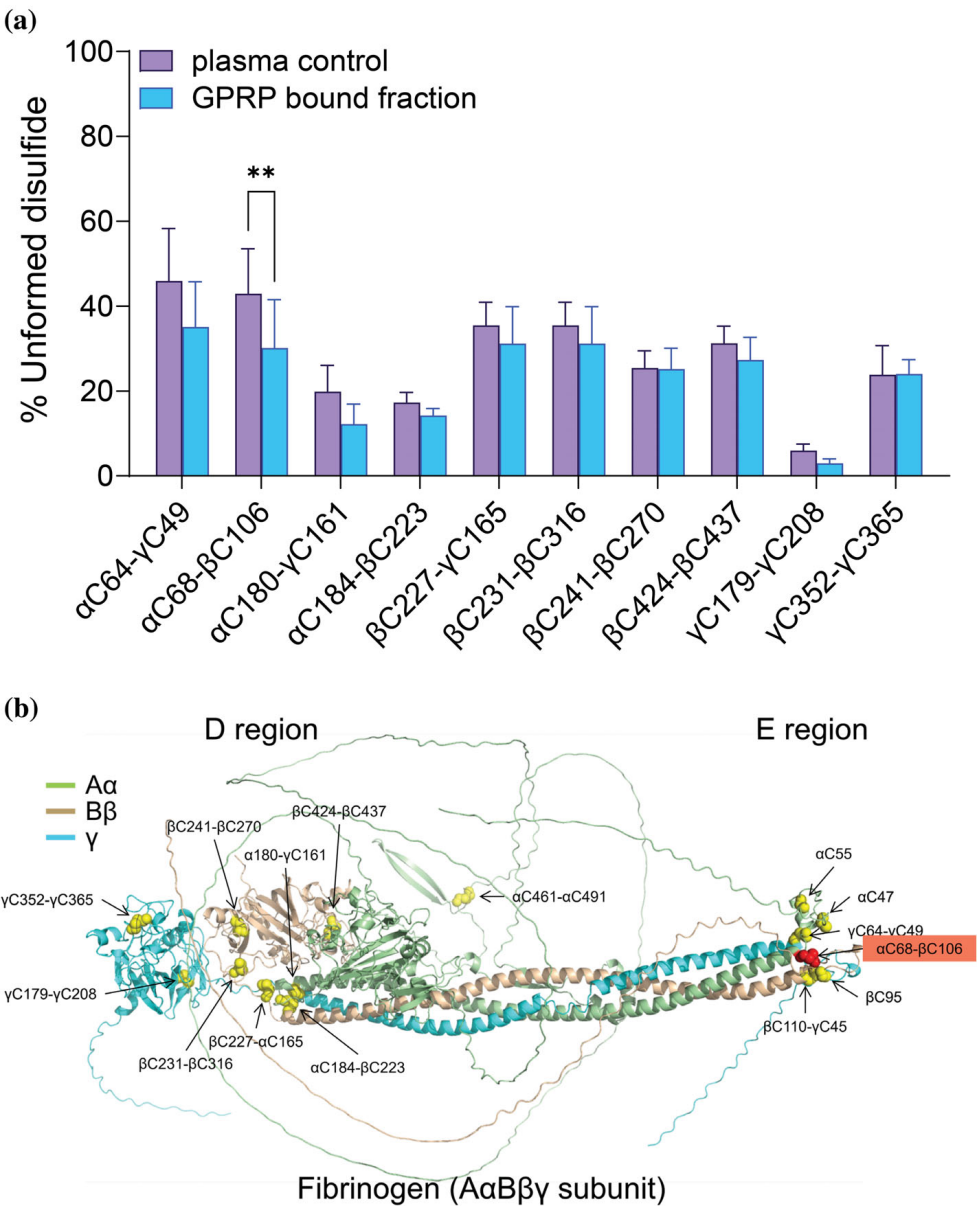
A γ‐nodule ligand does not select for different disulfide‐bonded fibrinogen states. (a) Redox states of 10 of the 12 plasma fibrinogen disulfide bonds captured on GPRP‐coated beads. The bars and errors represent the mean ± SD of six different healthy human plasma samples. Two‐way ANOVA with Tukey's multiple comparison tests were performed. **p < 0.01. (b) The AlphaFold3 ribbon structure of the fibrinogen subunit. The α chain is light green, β chain is wheat and γ chain is cyan. The 12 intrachain and interchain disulfide bonds are indicated as yellow spheres. Only the E region αC68‐βC106 disulfide bond (in red) was significantly more formed in fibrinogen states captured by GPRP peptide.

The redox states of 10 intrachain and interchain disulfide bonds in plasma fibrinogen molecules captured on GPRP‐coated beads. Only one disulfide was significantly more formed in the GPRP‐bound molecules, the E region αC68‐βC106 bond (Figure 3). This contrasts with the results using the polyclonal antibody and GHRP ligands.

The B:b interaction contributes to lateral aggregation of fibrin protofibrils by mediating the binding of the GHRP “knob” exposed on the β chain of one fibrin monomer to the complementary “hole” located within the β‐nodule of another fibrin molecule. The enrichment of fibrinogen molecules with more formed β chain disulfides in the GHRP‐bound fraction suggests that the covalent status of these bonds influences the structure of the β chain “hole” and its capacity to engage the peptide ligand. Whether one or more β‐nodule disulfide bonds dominate this process was examined by in silico analysis of the disulfide‐bonded states of the fibrinogen β chain.

### 2.5 An unformed βC424–βC437 disulfide is predicted to increase local structural flexibility of the D region β chain

The fibrinogen β‐nodule contains five intrachain or interchain disulfide bonds (Figure S2). If all 5 bonds can exist in formed or unformed states, which our results support, and that there is no special dependency on bond formation, which is not known, the β chain can exist in a maximum of 32 (Wolberg, 2023) possible disulfide‐bonded states (Figure S2). To investigate the structural implications of these different states, we employed AlphaFold3 to systematically predict structures corresponding to each disulfide‐bonded state via sequential in silico mutation of disulfide bond cysteines to alanines.

**FIGURE 4:**
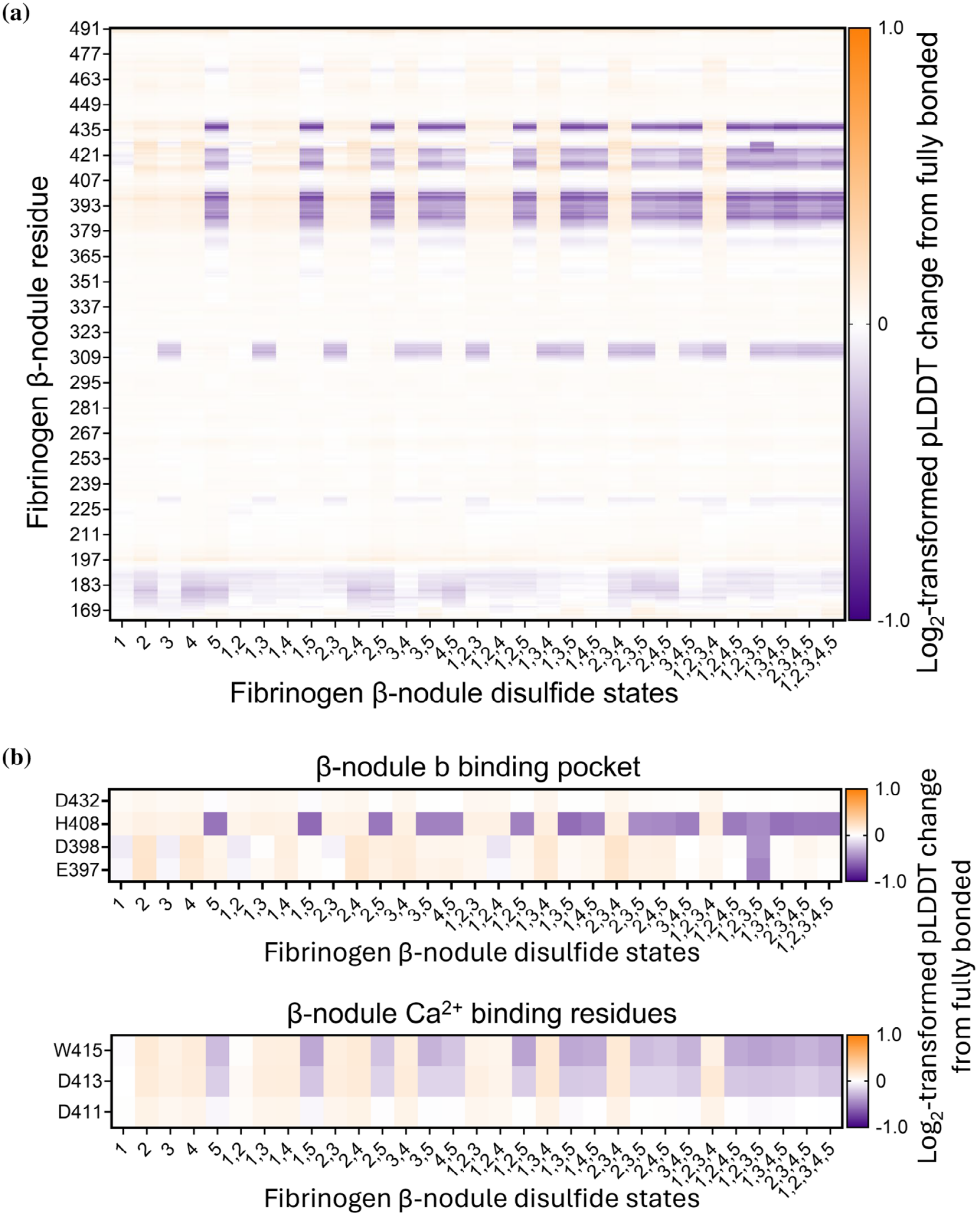
An unformed βC424‐βC437 disulfide is predicted to increase local structural flexibility of the β‐nodule. (a) Positions of five fibrinogen β chain disulfide bonds in the D region as predicted by AlphaFold3. The average percentage with which each bond is missing as per Figure 2. (b) All 32 theoretically possible disulfide states of this part of the fibrinogen molecule. (c) pLDDT scores per residue (*y*‐axis) were extracted from AlphaFold3 predicted structures and plotted relative to the fully bonded structure after Log2 transformation for normalization purposes. A decrease in pLDDT score compared to fully bonded structure (purple) indicates a predicted increase in structural flexibility, whereas an increase (orange) indicates a predicted decrease in flexibility. (d) The same data is shown for the residues comprising the β‐nodule binding pocket and Ca^2+^‐binding residues.

Intrinsic structural flexibility of the 32 different disulfide states of the fibrinogen β chain was inferred using pLDDT scores, an emerging proxy for assessing local structural flexibility within protein models (Guo et al., 2022; Manalastas‐Cantos et al., 2024; Wroblewski & Kmiecik, 2024). Log2‐transformed fold‐changes in amino acid pLDDT scores of the 31 different disulfide states of the fibrinogen β chain compared to the fully bonded structure were calculated (Figure 4a). A reduction in pLDDT score (purple color) compared to fully bonded structure indicates an increase in predicted structural flexibility and a comparative increase (orange color) predicts a decrease in flexibility in the amino acids indicated on the y‐axis. This analysis revealed the critical role of the βC424–βC437 disulfide bond (bond 5), where its absence significantly increased the predicted structural flexibility of the β‐nodule. Specifically, loss of this bond notably increased flexibility around residues constituting the GHRP binding pocket (E397, D398, H408, and D432) and nearby Ca^2+^ ion‐binding sites (D411, D413, and W415) (Everse et al., 1998) (Figure 4b). In contrast, the absence of one or more of the other four disulfide bonds generally had no or minor additive effects on flexibility.

### 2.6 An unformed βC424–βC437 disulfide is predicted to decrease B:b binding affinity

**FIGURE 5:**
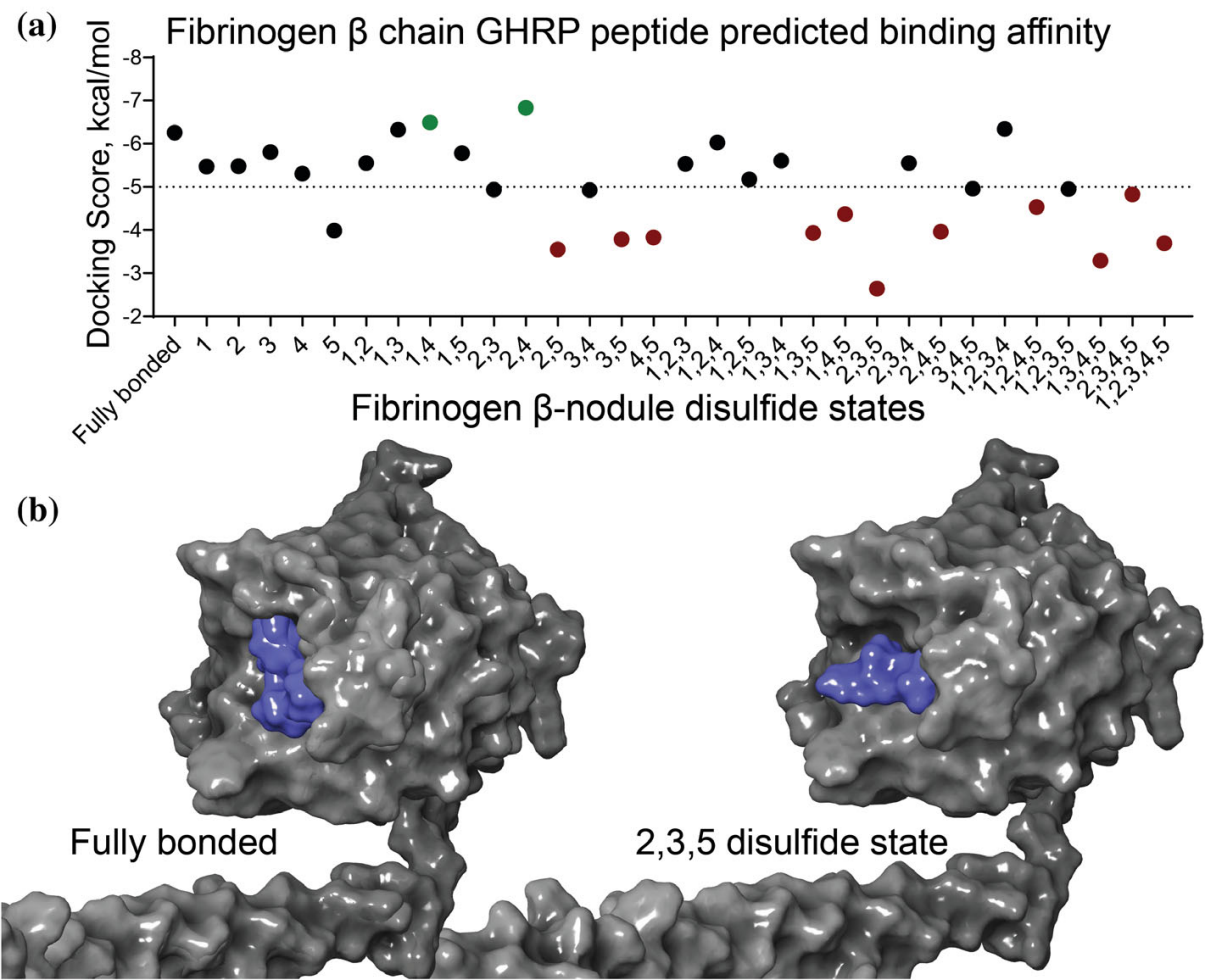
An unformed βC424–βC437 disulfide is predicted to decrease B:b binding affinity. (a) The absence of bond 5 (C424–C437) causes an increase in GHRP binding pocket flexibility that results in a predicted decreased in GHRP peptide binding affinity. An arbitrary Docking Score cut‐off of −5 kcal/mol was chosen to differentiate predicted “good” and “bad” interactions. (b) Representative docking outcomes of the most energetically favorable position of GHRP is shown for the fully bonded β‐nodule and for the disulfide state lacking bonds 2, 3, and 5 for comparison. Surface area of β chain is shown in grey and peptide in blue.

To further explore the functional impact of fibrinogen β chain disulfide states on the B:b interaction, Schrödinger's Maestro was utilized to predict binding affinities of the GHRP peptide to the β‐nodule. Absence of the βC424–βC437 disulfide bond (bond 5) markedly decreased the binding affinity for the GHRP peptide, demonstrated by an increased docking score (Figure 5). An arbitrary cutoff of −5 kcal/mol differentiated “good” binders (fully bonded and disulfide mutant states 2 and 4) from “poor” binders (disulfide mutants 5 and 2,3,5).

## 3 DISCUSSION

When probabilities of bond formation are taken into account (Hogg, 2020), it is clear that disulfide bond formation in fibrinogen is dependent on the state of other bonds, which reduces the number of possible states from the maximum unconditional 2048 based on the 11 disulfides measured in our analyses. The conditional nature of disulfide bond formation in fibrinogen is not known, although as the γC179–γC208 bond is almost always formed (fraction formed is 0.912) we speculate that formation of one or more other bonds is dependent on the γC179–γC208 being formed. The abundance of fibrinogen disulfide‐bonded states was estimated by collecting plasma fibrinogen molecules on beads coated with three different fibrinogen ligands.

Polyclonal anti‐fibrinogen antibodies resolved states where disulfides across the molecule were less formed, a β‐nodule ligand resolved states in which β‐nodule and central E region disulfides were more formed, while a γ‐nodule ligand resolved states in which only one disulfide was more formed. This result is inconsistent with the presence of only a few fibrinogen states where many bonds in individual molecules are either all formed or all unformed. The polyclonal antibody results point to states where several individual bonds are less formed in molecules, while the peptide binding results point to the opposite. In particular, the molecules that selectively bind the β‐nodule ligand are more formed in some areas of the molecule but not others. These findings support the existence of a large pool of fibrinogen disulfide‐bonded states with different conformations that selectively bind different ligands.

N‐linked glycosylation of the β and γ chains and oxidative modifications of susceptible residues have been reported for fibrinogen (Nencini et al., 2024). The extent to which such modifications are mechanistically coupled to specific disulfide states remains largely undefined, although there is currently no evidence that post‐translational modifications determine or influence disulfide‐bonded state. In the present study, we did not observe systematic differences in non‐cysteine modifications between ligand‐selected fractions within the limits of detection. We also found no evidence for dynamic changes in the disulfide states of fibrinogen under the conditions of our experiments. For example, we did not observe the formation of non‐native or mixed disulfide bonds with small molecule thiols.

The finding that polyclonal antibodies preferentially bind subsets of fibrinogen disulfide‐bonded states has potentially significant implications for the use of these reagents generally. Polyclonal antibodies are employed extensively to characterize their protein antigens in many experimental settings, including measuring protein concentration and function in vitro, ex vivo and in fixed and living tissues. Our findings indicate that consideration should be given to what disulfide‐bonded antigen states are recognized by different polyclonal antibodies. For instance, the distribution of disulfide‐bonded states of certain antigens in some individuals may be altered due to genetic or environmental factors. Some antigen populations may be preferentially recognized by the antibodies, for instance, and so bias the interpretation of results.

Fibrinogen is the soluble precursor of fibrin that polymerizes at injury sites to stabilize the developing thrombus. We have reported that the disulfide bonds in polymerized fibrin are globally more formed than in the parent fibrinogen and have suggested that this is due to disulfide bond formation in the fibrin (Butera & Hogg, 2020). The β‐nodule interactions with GHRP ligands on other fibrin molecules contribute to lateral aggregation of fibrin protofibrils (Weisel & Litvinov, 2013). We show herein that the GHRP ligand preferentially binds fibrinogen states in which seven β‐nodule and central E region disulfides are more formed and that the ligand does not induce disulfide bond formation in fibrinogen. This finding supports selection of states with more formed disulfides during fibrin polymerization, rather than formation of disulfides in the fibrin polymer.

To examine the link between disulfide state and conformation, we modeled all 32 possible disulfide‐bonded combinations of the β‐nodule using AlphaFold3. The traditional method of studying the roles of disulfide bonds in proteins is via mutagenesis, where the disulfide bond is ablated by replacing the Cys with Ala or Ser and the consequences for the structure and/or function of the recombinant protein are measured. This is problematic when a protein contains many partially formed disulfide bonds. For example, eliminating one disulfide bond may affect the redox state of other bonds in the protein and so introduce an artifact, or mutagenesis of one or more bonds may prevent proper maturation and thus analysis of the protein. Another approach is molecular dynamics studies that can become impractical in terms of time and cost when several disulfide bond states need to be considered, such as the 32 possible disulfide‐bonded states of the β‐nodule.

While both crystal structures and AlphaFold3 structural models reveal a snapshot of what is, in most cases, a highly dynamic protein structure, they can still offer valuable information on a protein's form‐function relationships. The pLDDT score generated by AlphaFold3 has emerged as a useful proxy for assessing local structural flexibility within protein models. High pLDDT values typically correspond to well‐ordered, rigid regions, whereas lower scores often indicate intrinsically flexible or disordered regions. Recent studies have validated the correlation between pLDDT scores and experimentally determined measures of protein dynamics, such as B‐factors and NMR‐derived order parameters, supporting the use of pLDDT as a surrogate for protein flexibility in silico (Abramson et al., 2024; Akdel et al., 2022; Al‐Masri et al., 2023; Guo et al., 2022; Ma et al., 2023; Manalastas‐Cantos et al., 2024; Saldano et al., 2022).

Our analysis indicated that the conformational flexibility of the β‐nodule depends predominantly on the oxidation state of a single disulfide, βC424–βC437. Loss of this bond increased the predicted mobility of residues forming the GHRP‐binding pocket and the adjacent calcium‐binding site, whereas the absence of other β‐nodule disulfides had minimal additive effects. The reduced rigidity associated with an unformed βC424–βC437 bond also corresponded to a predicted decrease in GHRP binding affinity. These results suggest that the βC424–βC437 disulfide stabilizes the β‐nodule conformation required for ligand engagement.

Recent structural analyses have emphasized the dynamic nature of fibrinogen, with both experimental and computational studies demonstrating substantial interdomain motion and conformational plasticity in solution (Medeiros et al., 2025; Pinelo et al., 2023). The present findings extend this view by showing that variability in disulfide bonding is an important component of the molecule's conformational heterogeneity. Furthermore, the dependence of β‐nodule architecture on the βC424–βC437 disulfide bond illustrates how disulfide heterogeneity can modulate local flexibility and ligand affinity within a single protein population. Fibrinogen thus exemplifies how multiple disulfide‐bonded states can endow a protein with a continuum of conformations that coexist under physiological conditions. Such structural plasticity may confer adaptive advantages, enabling secreted proteins to sample conformational ensembles suited to different environments or binding partners.

## 4 MATERIALS AND METHODS

### 4.1 Blood collection and processing

All procedures involving collection of human blood from healthy volunteers were in accordance with the Human Research Ethics Committee of the University of Sydney (approval HREC 2014/244) and informed consent was obtained from all individuals. Blood was collected by venipuncture using a 21 g butterfly Terumo needle from 6 healthy donors (3 females and 3 males, aged between 22 and 36) on no medications. The first 5 mL of blood was discarded to avoid any thrombin that may have been generated around the needle insertion site and then drawn into tubes containing 3.2% v/v sodium citrate. Plasma was prepared by twice centrifugation at 800*g* for 20 min at room temperature, after which it was aliquoted and snap‐frozen in liquid nitrogen. Plasma was stored at −80°C until use. When required, plasma was thawed at 37°C and diluted two times in PBS prior to recalcification by addition of 10 mM CaCl_2_ and 10 μM D‐phenylalanyl‐L‐prolyl‐L‐arginine chloromethyl ketone (Sigma) to inhibit thrombin activity.

### 4.2 Selection of a fibrinogen molecules using anti‐fibrinogen polyclonal antibodies

Dynabeads (2 mg, Life Technologies) were coated with 16 μg of polyclonal anti‐fibrinogen antibody (Dako, Cat. No. A0080) in 1 mL of phosphate‐buffered saline (PBS) on a rotating wheel for 1 h at 22°C and the excess antibody removed by washing three times with 1 mL of PBS. Plasma samples were incubated with 2 mg of coated beads on a rotating wheel for 1 h at 22°C. The beads were collected using a magnet and incubated in 0.3 mL of 4 mM 2‐iodo‐N‐phenylacetamide (^12^C‐IPA) in PBS for 1 h at room temperature with gentle rotation, while kept away from light. Control samples of 5 μL plasma were also incubated with 4 mM ^12^C‐IPA in the dark.

### 4.3 Selection of fibrinogen molecules using GHRP or GPRP peptides

GHRPLC and GPRPLC peptides were synthesized by Genscript. Thirty μM of peptide was incubated with 20 μL of maleimide agarose (Cube Biotech) in 0.5 mL of PBS with 1 mM ethylenediaminetetraacetic acid (EDTA) for 1 h with rotation on a wheel. Agarose was pelleted by centrifugation at 500*g* and washed with 0.5 mL of PBS three times prior to blocking for 1 h with 50 mM glutathione in PBS with 1 mM EDTA and three more washes with 0.5 mL of PBS. Control (no peptide) or peptide‐labeled agarose was centrifuged, resuspended in 500 μL of plasma and incubated for 1 h with rotation at room temperature. The agarose was centrifuged, the beads washed three times in 0.5 mL of PBS and resuspended in 0.5 mL of 4 mM 2‐iodo‐N‐phenylacetamide (^12^C‐IPA) in PBS for 1 h at room temperature with gentle rotation in the dark. Control samples of 5 μL plasma were also incubated with 4 mM ^12^C‐IPA in the dark. In some cases, 10 μL of plasma was incubated with 280 μM, 560 μM, or 1.12 mM GHRP peptide in a total of 25 μL for 30 min.

### 4.4 Quantification of the redox state of fibrinogen disulfide bonds

The bead supernatants were aspirated, and all samples were incubated with NuPAGE LDS sample buffer (Life Technologies) containing a further 4 mM ^12^C‐IPA for 30 min at 60°C. Samples were resolved on SDS‐PAGE at 150 V for 65 min before gels were stained with colloidal Coomassie (Sigma) and destained in 7% acetic acid and 10% methanol. An example Coomassie gel is shown in Figure S1. Fibrinogen bands were excised, diced into 1 mm^3^ slices, destained in 25 mM NH_4_HCO_3_ with 50% acetonitrile, dried in 100% acetonitrile, incubated with 40 mM dithiothreitol and washed. The gel slices were incubated with 5 mM ^13^C‐IPA (Cambridge Isotopes) in 25 mM NH_4_HCO_3_ with 10% DMSO for 1 h at room temperature in the dark to alkylate the disulfide bond cysteines. Gel slices were washed and dried as above before digestion of proteins with 35 μL of 12.5 ng/μL trypsin (Promega, V5280, Trypsin Gold, Mass spectrometry Grade) in 25 mM NH_4_HCO_3_ overnight at 30°C. Peptides were eluted from the slices with 5% formic acid and 50% acetonitrile.

Liquid chromatography, mass spectrometry and data analysis were performed as previously described (Al‐Masri et al., 2023). Briefly, peptides were analyzed on a Thermo Fisher Scientific Ultimate 3000. Two hundred ng of peptide was injected and resolved on a 35 cm × 75 μm C18 reverse phase analytical column with integrated emitter using a 2%–35% acetonitrile over 20 min with a flow rate of 250 nL/min. The peptides were ionized by electrospray ionization at +2.0 kV. Tandem mass spectrometry analysis was carried out on a Q‐Exactive Plus mass spectrometer using HCD fragmentation. The data‐dependent acquisition method acquired MS/MS spectra of the top 10 most abundant ions with charged state ≥2 at any one point during the gradient. MS/MS spectra were searched against the Swissprot reference proteome using Mascot search engine (Version 3.1, Matrix Science). Precursor mass tolerance and fragment tolerance were set at 10 ppm and the precursor ion charge state to 2+ and 3+. Variable modifications were defined as oxidized Met, deamidated Asn/Gln, N‐terminal pyro Glu/Gln, iodoacetanilide derivative cysteine and iodoacetanilide‐^13^C derivative cysteine with full trypsin cleavage and up to one missed cleavage. Only peptides with a peptide score > 30 (*p* < 0.05) and error <6 ppm were selected for quantification of relative abundance. Ion chromatograms of peptides labeled with ^12^C‐IPA and/or ^13^C‐IPA were generated using FreeStyle software (Thermo Fisher). The area under the curve (AUC) of each peptide was calculated using the automated peak detection function built into FreeStyle. The fraction of unformed disulfide bond was calculated as the AUC of ^12^C‐IPA labeled peptide divided by the AUC of ^12^C‐IPA plus ^13^C‐IPA labeled peptides. The data was routinely searched for peptides containing unlabeled cysteine thiols and these were not detected, which indicates that alkylation of unpaired cysteine residues by ^12^C‐IPA or ^13^C‐IPA was complete in the protein.

### 4.5 Structure prediction using AlphaFold3

The structure of all 32 possible disulfide‐bonded states of the fibrinogen D region was predicted using AlphaFold3. The canonical Uniprot sequences of the human fibrinogen Aα (P02671), Bβ (P02675), and γ (P02679) chains were used and sequential cysteine to alanine mutations were introduced to create 31 different disulfide mutants (Figure S2). Each structure prediction was made in the presence of two Ca^2+^ ions and seeded to 42. For each covalent state, the top‐ranked AlphaFold model (rank 0, as determined by the model confidence score) was used for analysis in PyMol. Across all 32 models, the D‐domain predictions exhibited uniformly high confidence, with mean predicted Local Distance Difference Test (pLDDT) values of 85.76 ± 0.72, predicted Template Modeling (pTM) scores of 0.86 ± 0.008, and no extended regions of low‐confidence prediction. The pLDDT score was extracted and plotted Log2 transformed relative to the fully‐bonded structure. We note that the canonical Aα sequence used in these predictions corresponds to the αE splice variant, which contains an extended C‐terminal region that is not present in the predominant plasma isoform; this difference lies outside the D region and does not impact the β‐ or γ‐nodules studied here. Additionally, structural prediction of the fibrinogen D region using AlphaFold3, while delivering high structural similarity to the fibrinogen crystal structures (α‐carbon RMSD of 0.53 Å over 574 atoms), suggests differences in disulfide bond formation compared to experimentally determined structures. The disulfide bond pairings are compared in Table S1.

### 4.6 Docking analysis

All predicted protein structures were prepared using the Protein Preparation Wizard in Schrödinger's Maestro. Hydrogens were added, and the protonation states of residues were adjusted to pH 7.4 using Epik. The peptide ligand was prepared using a combination of LigPrep and ConfGen. LigPrep was used to generate a set of possible tautomeric and protonation states at pH 7.4, ensuring chemical diversity in the ligand conformations. The ligand was then subjected to ConfGen to generate an ensemble of low‐energy conformers, capturing potential structural flexibility. Protein and peptide conformations were minimized using the OPLS3e force field.

A docking grid was generated to define the binding site on the protein where the peptide is predicted to bind. The grid was centered around four key binding residues in the β chain (E427, D428, H438, and D462) (Everse et al., 1998). A cubic grid box was set to encompass the entire binding region, with flexibility to accommodate the peptide's maximum size of 14 Å. Default settings were used to allow optimal sampling within the defined site.

Peptide–protein docking was performed using Glide's peptide docking protocol. The Docking Score scoring function was employed to rank binding poses based on predicted binding affinity. Ligand flexibility was allowed during docking, with the sampling options set to consider various peptide conformations and orientations in the binding pocket. The most favorable peptide binding pose was selected based on the lowest Docking Score and used for comparison.

## AUTHOR CONTRIBUTIONS

**Aster E. Pijning:** Writing – original draft; investigation; data curation; conceptualization; formal analysis; writing – review and editing. **Diego Butera:** Investigation; methodology; writing – review and editing. **Philip J. Hogg:** Conceptualization; funding acquisition; writing – original draft; writing – review and editing; supervision; formal analysis.

## Supporting information


**TABLE S1.** Comparison of the disulfide bond pairing reported in the Uniprot description of fibrinogen and the bonds predicted by AlphaFold3.
**TABLE S2:** Fraction of fibrinogen disulfide bonds that are formed in populations of the plasma protein. The fraction formed values are the mean values of 3 different healthy human plasma samples (data from Figure 1a) and are equivalent to the probability that the disulfide bond is formed in the population of fibrinogen molecules.
**FIGURE S1:** Example SDS‐PAGE gel of GHRP‐ or GPRP‐bound plasma fibrinogen fractions. The 260 kDa molecular mass standard is shown at left.
**FIGURE S2:** Positions of the 5 fibrinogen β‐nodule disulfide bonds and the 32 possible disulfide‐bonded states. The AF3 ribbon structure of the fibrinogen β‐nodule. The α chain is light green, β chain is wheat and γ chain is cyan. The five intrachain and interchain disulfide bonds are indicated as yellow spheres.
**FIGURE S3:** Crystal structures (1) of the fibrinogen β‐ and γ‐nodules containing bound GHRP or GPRP peptides, respectively. The α chain is light green, β chain is wheat and γ chain is cyan. The β‐nodule βC424–βC437 and γ‐nodule γC352–γC365 disulfide bonds flanking the binding pockets are indicated as yellow spheres. The GHRP and GPRP peptides are shown as red sticks and calcium ions as orange spheres.
**FIGURE S4:** The β‐nodule ligand GHRP does not induce disulfide bond formation in fibrinogen. The redox state of the fibrinogen β‐nodule disulfide bonds in plasma fibrinogen before (plasma control) and after incubation with 2, 5, or 10‐fold the *K*_d_ of GHRP peptide (140 μM^7^). The bars and errors represent the mean ± SD of from 3 different healthy human plasma samples. two‐way ANOVA with Tukey's multiple comparison tests were performed. All comparisons were *p* > 0.05.

## Data Availability

The mass spectrometry proteomics data have been deposited to the ProteomeXchange Consortium via the PRIDE (Perez‐Riverol et al., 2022) partner repository with the dataset identifier PXD076459 and 10.6019/PXD076459. All other relevant data are available from the authors.
