# Supplementary material for: Multiple disulfide‐bonded states confer extensive conformational diversity in fibrinogen
Source: Protein Sci. 2026 Apr 6;35(5):e70558. doi: 10.1002/pro.70558 (PMC13051931; doi:10.1002/pro.70558)
Supplement: Supplementary file 1 — TABLE S1. Comparison of the disulfide bond pairing reported in the Uniprot description of fibrinogen and the bonds predicted by AlphaFold3. TABLE S2: Fraction of fibrinogen disulfide bonds that are formed in populations of the plasma protein. The fraction formed values are the mean values of 3 different healthy human plasma samples (data from Figure 1a) and are equivalent to the probability that the disulfide bond is formed in the population of fibrinogen molecules. FIGURE S1: Example SDS‐PAGE gel of GHRP‐ or GPRP‐bound plasma fibrinogen fractions. The 260 kDa molecular mass standard is shown at left. FIGURE S2: Positions of the 5 fibrinogen β‐nodule disulfide bonds and the 32 possible disulfide‐bonded states. The AF3 ribbon structure of the fibrinogen β‐nodule. The α chain is light green, β chain is wheat and γ chain is cyan. The five intrachain and interchain disulfide bonds are indicated as yellow spheres. FIGURE S3: Crystal structures (1) of the fibrinogen β‐ and γ‐nodules containing bound GHRP or GPRP peptides, respectively. The α chain is light green, β chain is wheat and γ chain is cyan. The β‐nodule βC424–βC437 and γ‐nodule γC352–γC365 disulfide bonds flanking the binding pockets are indicated as yellow spheres. The GHRP and GPRP peptides are shown as red sticks and calcium ions as orange spheres. FIGURE S4: The β‐nodule ligand GHRP does not induce disulfide bond formation in fibrinogen. The redox state of the fibrinogen β‐nodule disulfide bonds in plasma fibrinogen before (plasma control) and after incubation with 2, 5, or 10‐fold the K d of GHRP peptide (140 μM7). The bars and errors represent the mean ± SD of from 3 different healthy human plasma samples. two‐way ANOVA with Tukey's multiple comparison tests were performed. All comparisons were p > 0.05. [file PRO-35-e70558-s001.docx]

**Supplementary Material**

**Multiple disulfide-bonded states confer extensive conformational diversity in fibrinogen**

Aster E. Pijning, Diego Butera, Philip J. Hogg

**Table S1.** Comparison of the disulfide bond pairing reported in the Uniprot description of fibrinogen and the bonds predicted by AlphaFold3.

| Uniprot bonds | | AF bonds | |
| --- | --- | --- | --- |
| Cys1 | Cys2 | Cys1 | Cys2 |
| αC47* | αC47 |  |  |
| αC55 | βC95 |  |  |
| αC64 | γC49 | αC64 | γC49 |
| αC68 | βC106 | αC68 | βC106 |
| αC180 | γC165 | αC180 | γC161 |
| αC184 | βC223 | αC184 | βC223 |
| αC461 | αC491 | αC461 | αC491 |
|  |  | αC632 | αC663 |
| αC799 | αC812 | αC799 | αC812 |
| βC110 | γC45 | βC110 | γC45 |
| βC227 | γC161 | βC227 | γC165 |
| βC231 | βC316 | βC231 | βC316 |
| βC241 | βC270 | βC241 | βC270 |
| βC424 | βC437 | βC424 | βC437 |
| γC34 | γC35 |  |  |
| γC179 | γC208 | γC179 | γC208 |
| γC352 | γ3C65 | γC352 | γC365 |

* The αC47-αC47 bond is in inter-molecular bond linking the two α-chains.

**Table S2**. Fraction of fibrinogen disulfide bonds that are formed in populations of the plasma protein. The fraction formed values are the mean values of 3 different healthy human plasma samples (data from **Fig. 1A**) and are equivalent to the probability that the disulfide bond is formed in the population of fibrinogen molecules.

| **Fibrinogen disulfide** | **Fraction formed** |
| --- | --- |
| αC64-γC49 | 0.526 |
| αC68-βC106 | 0.526 |
| βC110-γC45 | 0.658 |
| αC180-γC161 | 0.777 |
| αC184-βC223 | 0.830 |
| βC227-γC165 | 0.585 |
| βC231-βC316 | 0.585 |
| βC241-βC270 | 0.697 |
| βC424-βC437 | 0.651 |
| γC179-γC208 | 0.912 |
| γC352-γC365 | 0.700 |


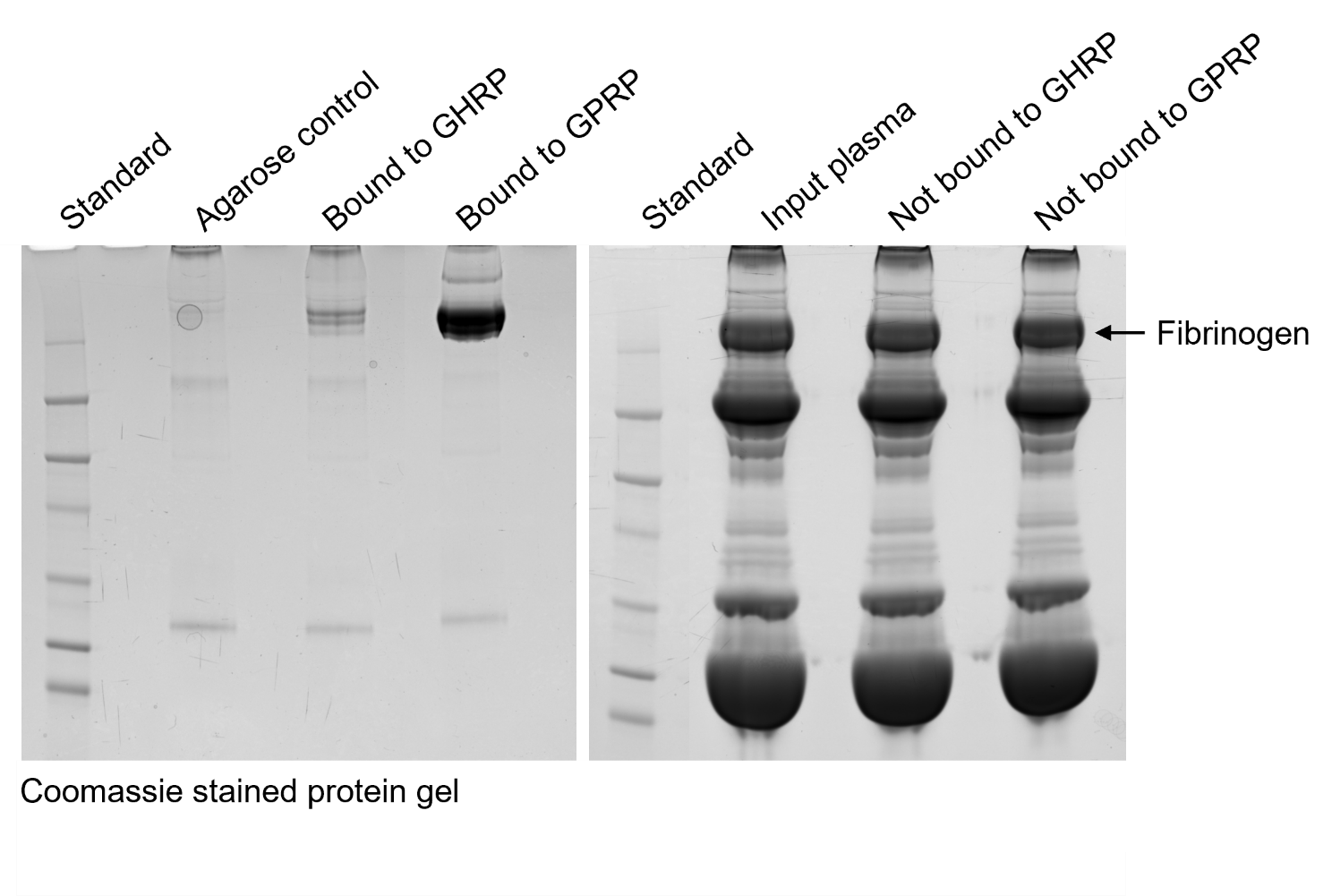


**Figure S1**. Example SDS-PAGE gel of GHRP- or GPRP-bound plasma fibrinogen fractions. The 260 kDa molecular mass standard is shown at left.


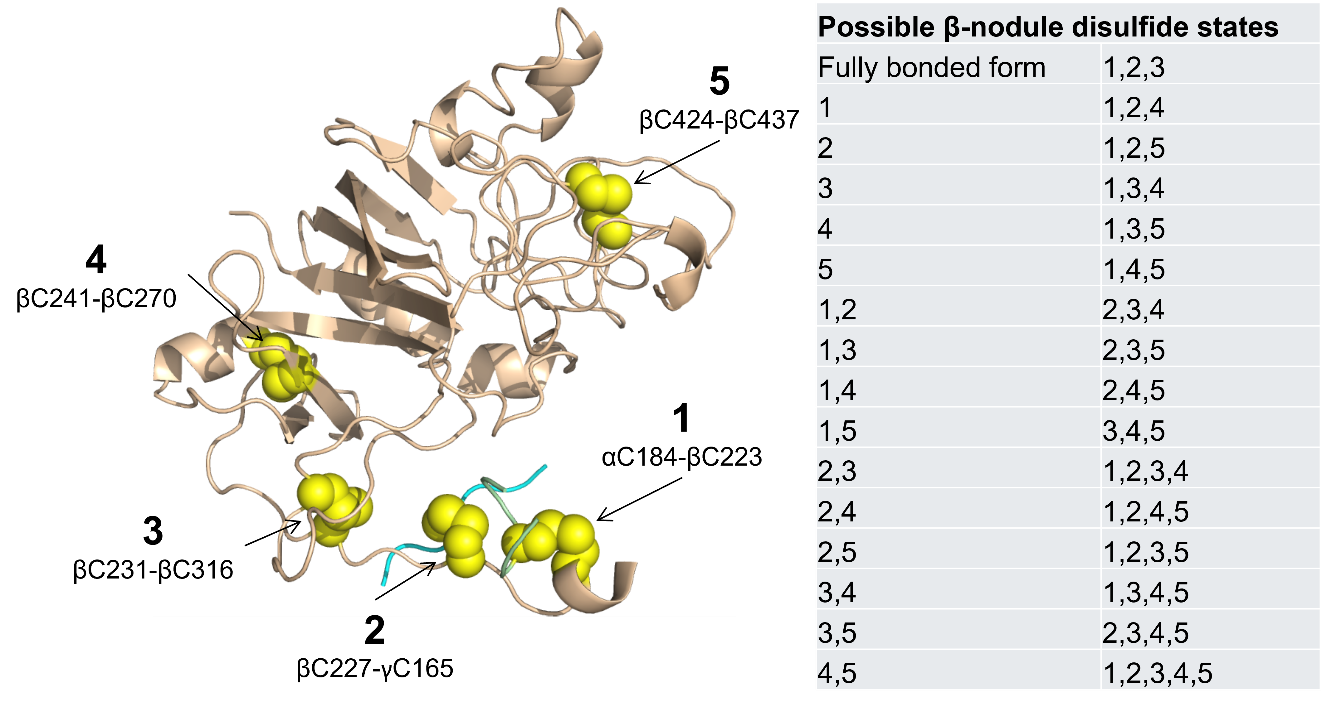


**Figure S2**. Positions of the 5 fibrinogen β-nodule disulfide bonds and the 32 possible disulfide-bonded states. The AF3 ribbon structure of the fibrinogen β-nodule. The α chain is light green, β chain is wheat and γ chain is cyan. The 5 intrachain and interchain disulfide bonds are indicated as yellow spheres.


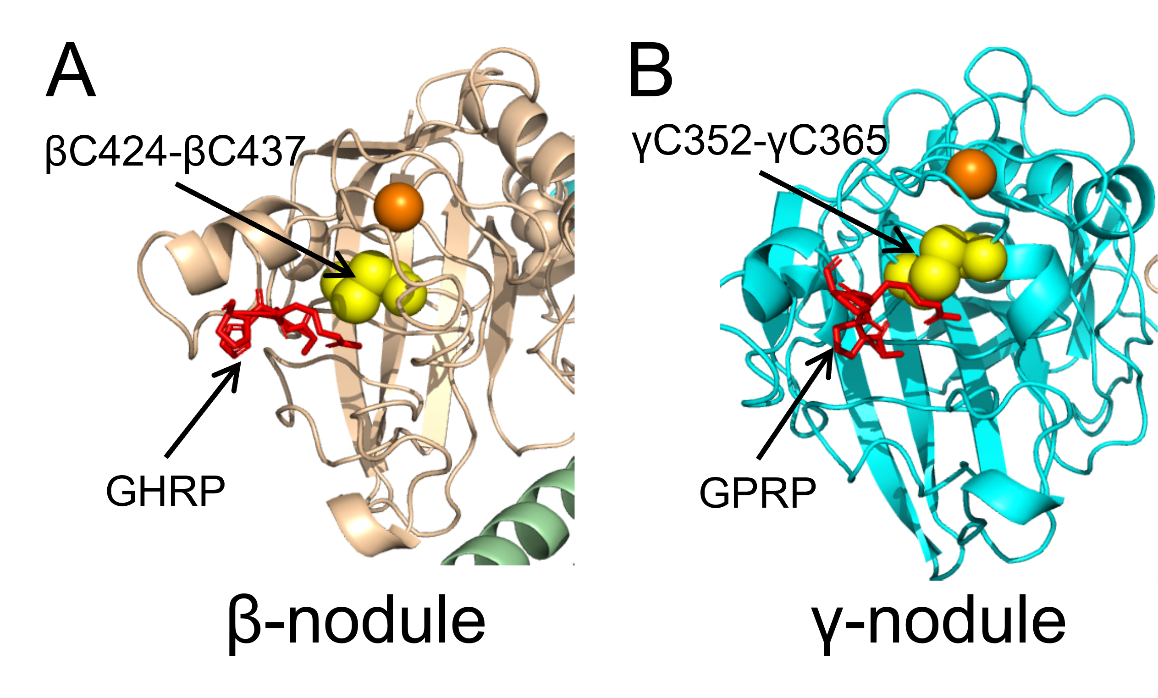


**Figure S3**. Crystal structures (1) of the fibrinogen β- and γ-nodules containing bound GHRP or GPRP peptides, respectively. The α chain is light green, β chain is wheat and γ chain is cyan. The β-nodule βC424-βC437 and γ-nodule γC352-γC365 disulfide bonds flanking the binding pockets are indicated as yellow spheres. The GHRP and GPRP peptides are shown as red sticks and calcium ions as orange spheres.

**Figure S4.** The β-nodule ligand GHRP does not induce disulfide bond formation in fibrinogen. The redox state of the fibrinogen β-nodule disulfide bonds in plasma fibrinogen before (plasma control) and after incubation with 2, 5, or 10-fold the Kd of GHRP peptide (140 µM^7^). The bars and errors represent the mean ± SD of from 3 different healthy human plasma samples. 2-way ANOVA with Tukey’s multiple comparison tests were performed. All comparisons were p > 0.05.

**References**

1. Everse SJ, Spraggon G, Veerapandian L, Riley M, Doolittle RF (1998) Crystal structure of fragment double-D from human fibrin with two different bound ligands. Biochemistry 37:8637-8642. PMID: 9628725 {Medline}
